# Benzydamine Reverses TMexCD-TOprJ-Mediated High-Level Tigecycline Resistance in Gram-Negative Bacteria

**DOI:** 10.3390/ph14090907

**Published:** 2021-09-07

**Authors:** Ziwen Tong, Tianqi Xu, Tian Deng, Jingru Shi, Zhiqiang Wang, Yuan Liu

**Affiliations:** 1 College of Veterinary Medicine, Yangzhou University, Yangzhou 225009, China; tongzw2021@gmail.com (Z.T.); xutianqi2021@gmail.com (T.X.); dt20210717@gmail.com (T.D.); shijr2021@gmail.com (J.S.); 2 Jiangsu Co-Innovation Center for Prevention and Control of Important Animal Infectious Diseases and Zoonoses, Joint International Research Laboratory of Agriculture and Agri-Product Safety, The Ministry of Education of China, Yangzhou University, Yangzhou 225009, China; 3 Institute of Comparative Medicine, Yangzhou University, Yangzhou 225009, China

**Keywords:** tigecycline resistance, efflux pump, *tmexCD-toprJ*, benzydamine, Gram-negative bacteria

## Abstract

Recently, a novel efflux pump gene cluster called *tmexCD1-toprJ1* and its variants have been identified, which undermine the antibacterial activity of tigecycline, one of the last remaining options effective against multidrug-resistant (MDR) Gram-negative bacteria. Herein, we report the potent synergistic effect of the non-steroidal anti-inflammatory drug benzydamine in combination with tigecycline at sub-inhibitory concentrations against various *temxCD-toprJ*-positive Gram-negative pathogens. The combination of benzydamine and tigecycline killed all drug-resistant pathogens during 24 h of incubation. In addition, the evolution of tigecycline resistance was significantly suppressed in the presence of benzydamine. Studies on the mechanisms of synergism showed that benzydamine disrupted the bacterial proton motive force and the functionality of this kind of novel plasmid-encoded resistance-nodulation-division efflux pump, thereby promoting the intracellular accumulation of tigecycline. Most importantly, the combination therapy of benzydamine and tigecycline effectively improved the survival of *Galleria mellonella* larvae compared to tigecycline monotherapy. Our findings provide a promising drug combination therapeutic strategy for combating superbugs carrying the *tmexCD-toprJ* gene.

## 1. Introduction

Antibiotic resistance mediated by chromosome mutation or horizontal gene transfer constitutes a global threat to public health [1,2]. Alarmingly, the emergence of carbapenem-resistant enterobacteriaceae (CRE) and MCR-producing enterobacterales (MCRPE) has aggravated the resistance crisis and leaves clinicians few choices from the existing antibiotic pipeline [3,4]. Tigecycline (TIG), a semisynthetic parenteral glycylcycline, was discovered in 1993 and introduced into clinical use in 2005 [5]. Notably, TIG has been considered as an extremely important treatment option for serious infections caused by multidrug-resistant (MDR) Gram-negative pathogens, including CRE and MCRPE [6]. However, the identification and prevalence of plasmid-mediated *tet*(X3/X4) [7] and the RND (resistance-nodulation-division) efflux pump gene cluster, *tmexCD1-toprJ1* [8,9], in clinically important pathogens are undermining the efficacy of TIG in clinical practice.

Generally, the mechanisms of tetracycline resistance in bacteria are commonly associated with tetracycline-specific efflux pumps, ribosomal protection, tetracycline-inactivating enzymes and multidrug-resistant efflux pumps [10]. Tet(X) and its orthologs are 388 amino acid flavin-dependent monooxygenases that can hydroxylate C11a of the tetracycline scaffold [11]. The *tet*(X3/X4) gene-encoded tetracycline-inactivating enzymes can confer high levels of TIG resistance to bacteria and are even able to inactivate all tetracyclines [12]. Furthermore, the RND family exporters, as a representative of MDR pumps, also made an important contribution to tigecycline resistance. In subcellular organization, the chromosomally encoded tripartite complex of RND efflux pumps are located in the inner membrane (IM) but contacted with the membrane fusion protein (MFP) and outer membrane (OM) channel [13], such as AcrAB-TolC in *E. coli* and MexAB-OprM in *P. aeruginosa*. Because the chromosomes of most Gram-negative bacteria are able to meet the requirement of RND superfamily efflux genes, the RND-type efflux pumps rarely appear on mobile genetic elements such as plasmids [14]. Unfortunately, the first identification of a plasmid-borne RND family multidrug efflux pump gene, *tmexCD1-toprJ1*, which confers transferable tigecycline resistance in *K. pneumoniae* challenges this notion; other mobile variants, including *tmexCD2-toprJ2* in *Raoultella ornithinolytica* and *tmexCD3-toprJ3* in *Proteus mirabilis*, were also subsequently reported [15,16]. Considering that conjugative plasmids play an important role in the spread of antimicrobial resistance, novel therapeutic strategies are urgently required to confront these tigecycline-resistant pathogens carrying the mobile RND-type pumps gene cluster.

Accordingly, repurposing approved drugs as potential antibiotic adjuvants offers a time and cost-effective means to combat MDR Gram-negative pathogens [17,18]. For example, our previous study indicated that metformin, an oral diabetes medicine, restored tetracycline activity against MDR bacteria by promoting intracellular accumulation of antibiotics and boosting immune response [19]. In addition, we found that the anti-HIV agent azidothymidine decreased Tet(X3/X4)-mediated bacterial resistance to tigecycline in *E. coli* by specifically inhibiting DNA synthesis and suppressing resistance enzyme activity [20]. However, the combination of azidothymidine and TIG displayed no direct synergistic activity against *tmexCD-toprJ*-positive Gram-negative bacteria. Benzydamine (BEN) is a locally acting non-steroidal anti-inflammatory drug and widely used pain-relieving and anti-inflammatory treatment for inflammatory conditions of the mouth and throat [21]. Recently, BEN has come to be considered a new drug for the treatment of bone disease due to its abilities to strengthen osteoblast differentiation and prevent bone loss [22]. However, its synergistic activity with antibiotics, particularly with tigecycline, is still not fully understood.

In this study, we reported that BEN markedly potentiated the antibacterial activity of tigecycline in the fight against *tmexCD-toprJ*-bearing bacteria, both in vitro and in the *Galleria mellonella* infection model. Furthermore, the combined use of benzydamine with tigecycline displayed potent bactericidal activity against *tmexCD-toprJ*-positive bacteria and suppressed the evolution of tigecycline resistance. The potentiation of BEN to TIG is attributed to the disruption of bacterial PMF and the dysfunction of the *tmexCD-toprJ*-mediated efflux pump. Our data demonstrate that the non-antibiotic agent benzydamine is a novel and potent antibiotic adjuvant in conjunction with tigecycline for the treatment of infection caused by *tmexCD-toprJ*-positive pathogens.

## 2. Results

### 2.1. Synergistic Activity between Benzydamine and Tigecycline against TMexCD-TOprJ-Positive Bacteria

To test the synergistic activity of BEN and TIG against TMexCD-TOprJ-positive pathogens, a collection of 12 clinical isolates of *K. pneumoniae* carrying a *tmexCD1-toprJ1*-bearing plasmid and two isolates of *P. mirabilis* carrying the chromosomal *tmexCD3-toprJ3* gene cluster, isolated from swine fecal samples taken in Jiangsu Province, were utilized [16]. Minimum inhibitory concentrations (MICs) of four classes of antibiotics, including meropenem, ciprofloxacin, colistin and tetracyclines, were determined using the standard broth micro-dilution method, with *E. coli* ATCC 25922 as control. The results showed that the MICs for BEN were 500 μg/mL in *K. pneumoniae* and 2000 μg/mL in *P. mirabilis* (Table 1). All TMexCD-TOprJ-positive Gram-negative bacteria exhibited high resistance to all tetracycline antibiotics and ciprofloxacin, but were susceptible to meropenem and colistin. Nevertheless, it was previously found that some pathogens harboring *tmexCD1-toprJ1* showed resistance to multiple antibiotics, including meropenem [23] and colistin [9,24]. With regard to TIG, the MIC values ranged from 16 to 32 μg/mL for these strains. Surprisingly, in the presence of one quarter of the MIC for BEN, the MIC values of TIG were significantly decreased, from by 4- to 256-fold, suggesting that the antibacterial activity of TIG was greatly potentiated in all test strains (Table 1). Notably, the potentiation of BEN to TIG was highly superior to the traditional efflux pump inhibitor 1-(1-naphthylmethyl)-piperazine (NMP) [15]. Meanwhile, it is plausible that the use of BEN would significantly reduce the administration dose of TIG, which may help alleviate potential side effects of TIG in the clinical setting.

**Figure 1:**
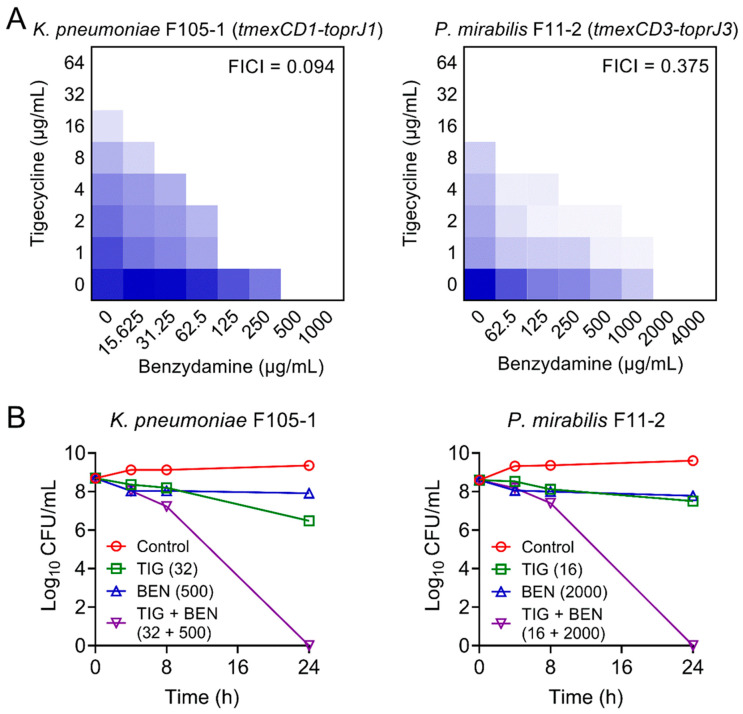
Synergistic activity of tigecycline and benzydamine against *temxCD-toprJ*-bearing Gram-negative bacteria. (**A**) Benzydamine effectively synergized with tigecycline against *temxCD1-toprJ1*-positive and *temxCD3-toprJ3*-positive pathogens by checkerboard assays. Dark blue regions represent higher cell density. Data represent the mean OD (600 nm) of three biological replicates. Synergy is defined as an FIC index of ≤0.5. (**B**) Killing activity of TIG plus BEN in LB media against *K. pneumoniae* F105-1 harboring *temxCD1-toprJ1* and *P. mirabilis* F11-2 harboring *temxCD3-toprJ3*. Data are representative of three biological replicates and presented as mean ± SD.

Furthermore, checkerboard assays were used to evaluate the synergism between BEN and TIG. This combination showed obvious synergistic activity with a fractional inhibitory concentration index (FICI) at 0.094 in *K. pneumoniae* F105-1 and 0.375 in *P. mirabilis* F11-2 (Figure 1A), suggesting a robust potentiation of BEN to TIG against plasmid-encoded TMexCD1-TOprJ1-expressing *K. pneumoniae* and intrinsic chromosomally-encoded TMexCD3-TOprJ3-positive *P. mirabilis*.

Next, we performed time-kill experiments to further investigate their synergistic bactericidal activity. Excitingly, the BEN plus TIG combination killed all pathogens with a dramatic reduction of bacterial loads by approximately 9-log_10_ after 24 h of incubation (Figure 1B). These results indicate that the combination of BEN and TIG displays potent bactericidal activity against various TMexCD-TOprJ-expressing Gram-negative bacteria.

### 2.2. BEN Prevents the Evolution of Tigecycline Resistance

**Figure 2:**
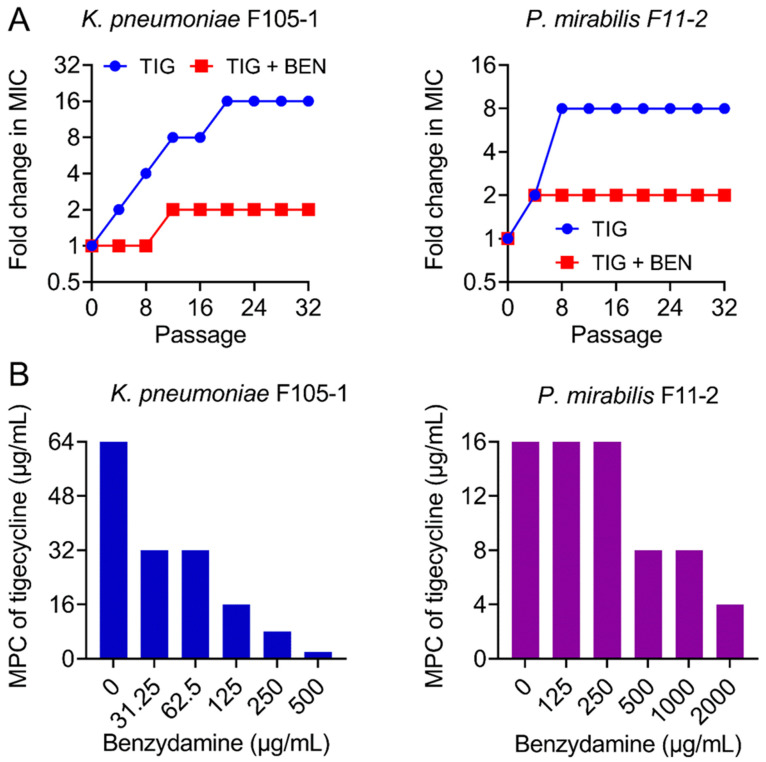
Benzydamine prevents the evolution of tigecycline resistance. (**A**) TIG resistance acquisition curves in the presence of sub-inhibitory concentration of TIG or a combination of BEN and TIG against novel *temxCD*-*toprJ*-harboring Gram-negative bacteria during 32 serial passages. (**B**) Mutant prevention concentrations (MPCs) of TIG in the presence of increasing concentrations of BEN against *temxCD*-*toprJ*-positive Gram-negative bacteria. Data are representative of three biological replicates.

The ideal antibiotic adjuvant should also meet some additional criteria, including the suppression of resistance development [25]. Hence, after discovering the strong synergism of BEN and TIG, we next explored whether BEN could prevent the evolution of TIG resistance. We performed serial passages of two TMexCD-TOprJ-positive bacteria with sub-MICs of TIG in the presence or absence of BEN (one quarter of MIC). TIG resistance levels were greatly elevated upon exposure to TIG, as its MIC values increased by 16-fold (*K. pneumoniae* F105-1) and 8-fold (*P. mirabilis* F11-2) over a period of 32 serial passages (Figure 2A). By contrast, the resistance level increased by only 2-fold in the combination treatment. In addition, mutant prevention concentrations (MPCs) of TIG in the presence of increasing concentrations of BEN were determined, which is an important factor for characterizing the capacity of bacteria to generate drug-resistant mutants [26]. Interestingly, the MPCs of TIG were significantly reduced from 64 to 2 μg/mL in *K. pneumoniae* F105-1 and from 16 to 4 μg/mL in *P. mirabilis* F11-2 (Figure 2B), indicating narrower mutation selection windows in the presence of BEN. These data clearly demonstrate that BEN restricts resistance evolution in TMexCD-TOprJ-positive bacteria.

### 2.3. BEN Deprives the Function of Efflux Pump by Dissipating Proton Motive Force

**Figure 3:**
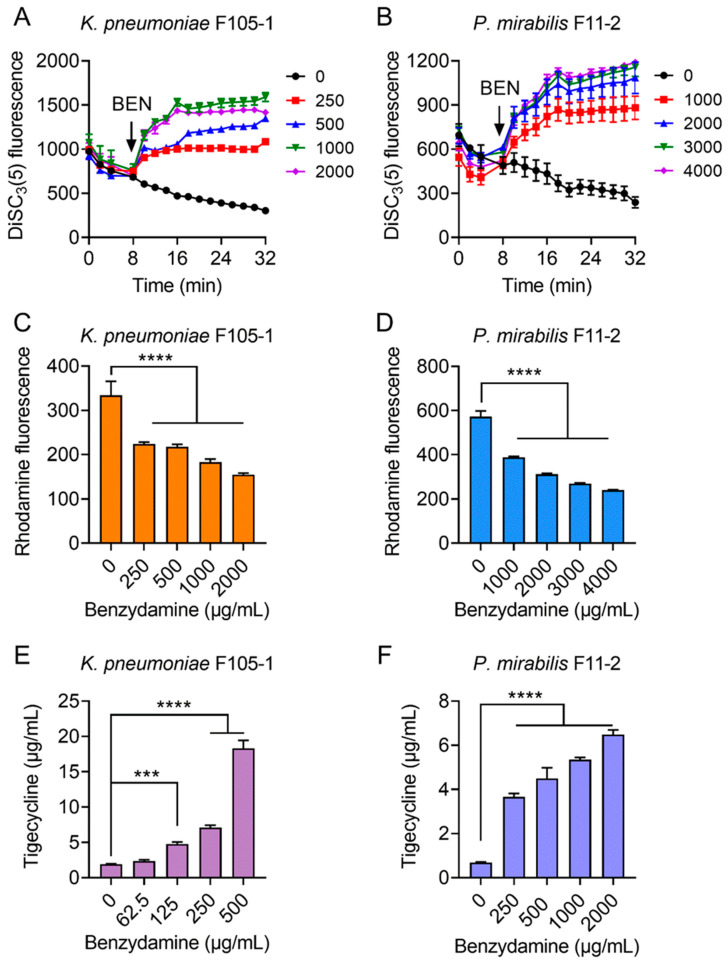
Tigecycline-benzydamine combination exerts synergy by dissipating bacterial proton motive force. (**A**,**B**) Membrane potential changes of in *K. pneumoniae* F105-1 (**A**) and *P. mirabilis* F11-2 (**B**) upon exposure to benzydamine, probed by potentiometric fluorophore DiSC3(5). (**C**,**D**) Function of efflux pump of *K. pneumoniae* F105-1 (**C**) and *P. mirabilis* F11-2 (**D**) carrying temxCD1-toprJ1 and temxCD3-toprJ3, respectively, after exposure to varying concentrations of benzydamine, measured by the fluorescence dye Rhodamine. (**E**,**F**) Intracellular accumulation of tigecycline in cells treated with benzydamine determined by LC-MS/MS analysis. Initial concentration of tigecycline was 32 μg/mL in *K. pneumoniae* F105-1 (**E**) and and 16 μg/mL in *P. mirabilis* F11-2 (**F**). All data are expressed as mean ± SD from three biological replicates and P values were determined by non-parametric one-way ANOVA (*** *p* < 0.001, **** *p* < 0.0001).

Considering that ATP synthesis is necessary for RND-type efflux pumps and that proton motive force (PMF) is the driving force for ATP production; thus, we hypothesized that BEN may dissipate bacterial PMF and thereby antagonize the efflux of TIG in TMexCD-TOprJ-positive bacteria. To test this hypothesis, we determined the PMF in bacteria after exposure to increasing concentrations of BEN by monitoring the fluorescence changes of DiSC_3_(5) [27]. PMF is composed of Δψ (the electrical potential) and ΔpH (the transmembrane difference) [28]. When the Δψ component of PMF is disrupted, DiSC_3_(5) is released from the cytoplasmic membrane to the extracellular milieu and fluorescence increases. Here, stationary-phase bacteria were stained with DiSC_3_(5) to measure fluorescence change and then cells were introduced to varying concentrations of BEN (0–2000 μg/mL for *K. pneumoniae* F105-1, 0–4000 μg/mL for *P. mirabilis* F11-2) (Figure 3A,B). Interestingly, the addition of BEN to probed cells caused increased fluorescence in a dose-dependent manner immediately, whereas the TIG alone showed no effect on the fluorescence, suggesting that BEN selectively disrupted the Δψ component of bacterial PMF.

It is suggested that PMF is essential for energy synthesis and material transportation [29,30]; thus, we next tested whether the damage to bacterial PMF will affect the efflux function of TMexCD-TOprJ and promote the intracellular accumulation of TIG. A rhodamine-based assay was used to assess the capability of this new RND-type efflux pump in pathogens after treatment with different concentrations of BEN (Figure 3C,D). As expected, dose-dependent reduced effluxes of rhodamine in *K. pneumoniae* F105-1 and *P. mirabilis* F11-2 were observed, as in the presence of BEN. Furthermore, we measured the intracellular concentration of TIG in bacteria after treatment with BEN by LC-MS/MS analysis. The results indicated that TIG had notably accumulated in cells under the actions of BEN (Figure 3E,F). In the absence of BEN, low levels of TIG in bacteria were detected. By contrast, the intracellular concentrations of TIG were markedly increased, by nearly 20-fold in *K. pneumoniae* F105-1 and 6-fold in *P. mirabilis* F11-2 when incubated with BEN at 1× MIC. Together, these results demonstrate that the potentiating mechanisms of BEN to TIG in TMexCD-TOprJ-positive bacteria can be attributed to the destruction of bacterial PMF, which inhibits the functions of *tmexCD-toprJ*-encoded efflux pumps and promotes intracellular accumulation of TIG.

### 2.4. BEN Restores In Vivo Efficacy of Tigecycline

**Figure 4:**
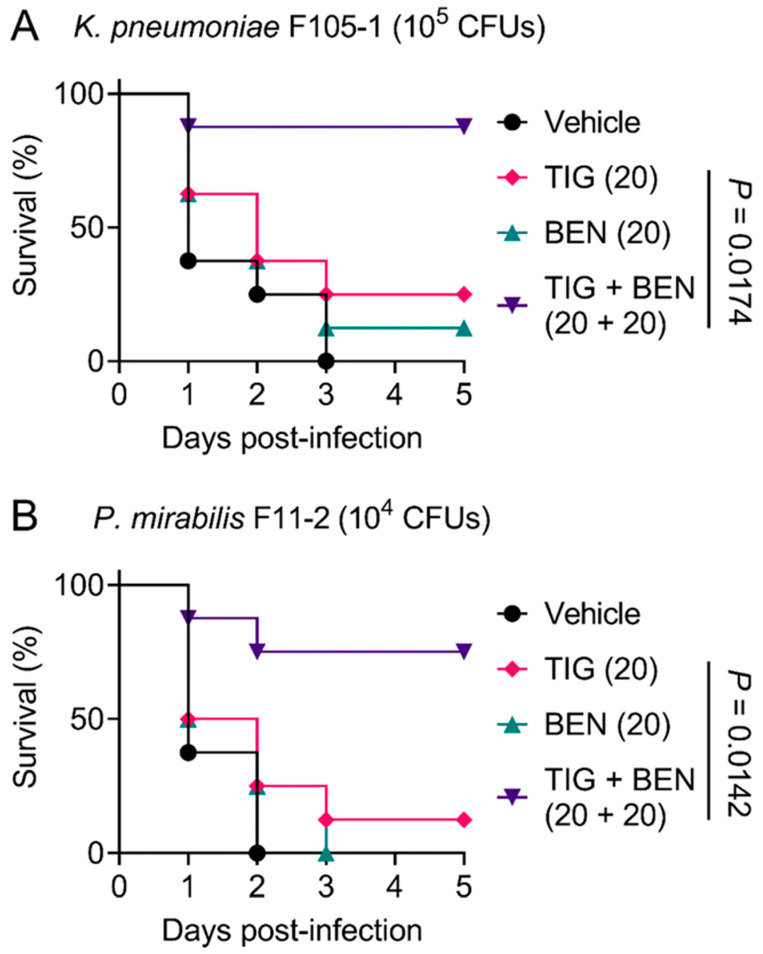
Benzydamine effectively improves tigecycline efficacy in *Galleria mellonella* infection model. Survival rates of *Galleria mellonella* larvae (*n* = 8 per group) infected by *K. pneumoniae* F105-1 (**A**) and *P. mirabilis* F11-2 (**B**) after treatment with PBS as vehicle, a single dose of benzydamine (BEN, 20 mg/kg), tigecycline (TIG, 20 mg/kg), or their combination. *p* values were determined by log-rank (Mantel–Cox) test.

In view of the robust synergistic effect of TIG and BEN in the fight against TMexCD-TOprJ-positive pathogens in vitro, we further explored the therapeutic potential of this drug combination therapy using a *Galleria mellonella* infection model. *Galleria mellonella* larvae were infected with a lethal dose of *K. pneumoniae* F105-1 (10^5^ CFUs) or *P. mirabilis* F11-2 (10^4^ CFUs). The infected larvae were then treated with a vehicle (PBS) control, BEN or TIG monotherapy, or a combination of BEN and TIG. For the vehicle control, all larvae died within three days. With regard to monotherapy, BEN and TIG treatment both led to a larvae survival rate of less than 25%. In contrast, a single dose of BEN plus TIG (20 + 20 mg/kg) significantly increased the survival rate of larvae compared to TIG monotherapy (*p* = 0.0174 or 0.0142, respectively) at five days post-infection (Figure 4A,B). Given that *K. pneumoniae* is the main cause of pneumonia, pyogenic liver abscess, endophthalmitis and other metastatic infections [31], the combination of BEN and TIG may provide an alternative regimen for *K. pneumoniae*-elicited infections. These data strongly confirm the in vivo efficacy of this drug combination in combating infections caused by TMexCD-TOprJ-positive pathogenic bacteria.

## 3. Discussion

As a result of bacterial resistance to carbapenems and colistin, TIG is recognized as the last antibiotic of choice for the treatment of complicated infections caused by MDR Gram-negative pathogens [32,33]. However, the identification of the mobile genetic element *tet*(X3/X4) and *tmexCD1-toprJ1* in clinically relevant pathogens from human and animal sources confers high levels of TIG resistance [8]. Notably, these novel TIG resistance genes are frequently located on the conjugative plasmids, which can transfer across intra- and inter-species, thus greatly accelerating the epidemic of antimicrobial resistance among various pathogens and posing a serious threat to public healthcare [34,35]. Alarmingly, there have been essentially no new classes of antibiotics or alternatives clinically approved in recent years. Thus, repurposing non-antibiotic agents as antibiotic adjuvants offers a promising strategy for addressing this troubling situation [36].

In this study, we found that the non-steroidal anti-inflammatory drug BEN reversed *tmexCD-toprJ*-mediated TIG resistance and remarkably potentiated the antibacterial activity of TIG against drug-resistant *K. pneumoniae* and *P. mirabilis*. Interestingly, although TIG is a bacteriostatic antibiotic, the combined use of BEN and TIG displayed potent bactericidal activity against TMexCD-TOprJ-positive bacteria in a time-dependent manner, highlighting the potency of this drug combination. The development and evolution of bacterial resistance to novel therapeutic strategies such as new antibiotics has been a huge challenge. Several previous studies reported that potent antibiotic adjuvants not only enhance antibiotic activity but also thwart the development of drug resistance [37,38]. Using similar assays, we demonstrated that the use of BEN substantially prevented the evolution of TIG resistance in TMexCD-TOprJ-positive bacteria and inhibited the enrichment of mutant subpopulations. Notably, the synergism potency of this drug combination has been verified in a *Galleria mellonella* infection model, which has been widely used to assess the therapeutic effectiveness of new antimicrobial agents owing to its various advantages, including convenience, low cost and a lack of ethical issues [39,40].

Investigations into the modes of action of BEN revealed that this compound dissipated the ΔΨ component of bacterial PMF and subsequently deprived the function of TMexCD-TOprJ-mediated novel RND-type efflux pumps, resulting in the intracellular accumulation of TIG. It is well known that TIG exhibits antibacterial activity by binding to the 30S ribosomal subunit and, finally, inhibiting protein synthesis [41]. Therefore, sufficient accumulation of TIG in cells is necessary for its actions. Interestingly, our findings showed BEN damaged the function of TMexCD-TOprJ efflux pumps by targeting bacterial PMF. Bacterial PMF play a crucial role in ATP synthesis, which in turn supports the energy source of efflux pumps [42]. Therefore, dissipating bacterial PMF contributes to combating efflux-mediated antibiotic resistance. Consistently, our previous study reported that metformin enhanced the antibacterial activity of doxycycline against *tet*(A)-positive pathogens by destroying the PMF in *E. coli* and promoting the intracellular uptake of doxycycline [19]. These findings strongly suggest that bacterial PMF may serve as a promising target for the identification of novel efflux pump inhibitors.

## 4. Materials and Methods

### 4.1. Minimum Inhibitory Concentration (MIC) Assays

MICs of all drugs were determined by the standard broth micro-dilution method in accordance with CLSI 2018 guidelines [43], using *E. coli* ATCC 25922 as the control. Generally, bacteria were cultured in LB broth overnight at 37 °C with 250 rpm sharking. All compounds were two-fold diluted in a 96-well microtiter plate (Corning, New York, NY, USA) containing 100 μL Mueller-Hinton broth (MHB, Qingdao Hope Bio-technology, Qingdao, China), followed by the addition of a prepared overnight bacteria culture (10^8^ CFUs/mL). After incubation at 37 °C for 18 h, the MIC value was defined as the lowest concentration of drugs with no visible growth of bacteria. Wells with MHB were only used as negative controls and wells with no drugs were used as positive controls. Experiments were performed with two biological replicates.

### 4.2. Checkerboard Assays

To verify the synergistic activity between TIG and BEN, the checkerboard assays were conducted [12]. First, 100 μL MHB was dispensed into a 96-well microtiter plate, and then the BEN and TIG were serially diluted at eight concentrations to establish an 8 × 8 matrix. Then, 100 μL stationary-phase bacterial suspensions of *K. pneumoniae* F105-1 or *P. mirabilis* F11-2 were added. The cultures were incubated at 37 °C for 18 h, and then the optical density of bacteria at 600 nm was measured by Microplate reader (Tecan, Männedorf, Switzerland). FIC index (FICI) was calculated according to the following formula: FICI = FIC_A_ + FIC_B_ = MIC_AB_/MIC_A_ +MIC_BA_/MIC_B_; FIC_A_ and FIC_B_ are the FIC index of drug A and B, respectively; MIC_A_ and MIC_B_ are the MIC of drug A and B, respectively; MIC_AB_ and MIC_BA_ are the MIC of one drug in combination with another. Synergism is defined with an FICI of ≤0.5. Experiments were conducted with biological replicates.

### 4.3. Time-Dependent Killing Studies

Overnight *K. pneumoniae* F105-1 and *P. mirabilis* F11-2 cells were diluted 1/1000 into LB broth at 37 °C for 5 h. Cells were then treated with BEN or TIG alone or their combination for 24 h. Aliquots of bacterial culture were removed at 0, 4, 8 and 24 h of incubations and resuspended in sterile phosphate buffer saline (PBS, 0.01 M, pH = 7.4). Finally, suspensions were plated on Luria–Bertani (LB) medium and incubated overnight at 37 °C. Bacterial numbers were calculated. The concentrations of the drugs used were 32 μg/mL TIG and 500 μg/mL BEN for *K. pneumoniae* F105-1 assay and 16 μg/mL TIG and 2000 μg/mL BEN for *P. mirabilis* F11-2 assay. LB broth with no drugs were used as a control. Experiments were performed with three biological replicates.

### 4.4. Resistance Development Assessment

The resistance development study was performed according to a previous study [44]. Briefly, overnight cultures of bacteria (*K. pneumoniae* F105-1 or *P. mirabilis* F11-2) were diluted 1/1000 into LB broth containing 0.25 × MIC of TIG or in combination with 0.25 × MIC of BEN. After incubation at 37 °C for 12 h, the MIC of the cultures was determined. Meanwhile, a 1/1000 dilution of the bacteria culture was performed into fresh medium supplemented with 0.25 × MIC of drugs for the next passages. This experiment was performed for 32 passages and the MIC increase of tigecycline was calculated.

### 4.5. Mutant Prevention Concentration (MPC) Determination 

The MPC assay was performed by a method described previously [45]. Bacteria (*K. pneumoniae* F105-1 or *P. mirabilis* F11-2) at 10^10^ CFUs were plated onto LB agar plates containing TIG alone or in combination with BEN at varying concentrations. Next, the plates were cultured in a 37 °C incubator for 72 h. The lowest concentration that restricted the growth of bacteria was defined as MPC. Experiments were performed with three biological replicates.

### 4.6. Proton Motive Force Assay

The proton motive force of *K. pneumoniae* F105-1 or *P. mirabilis* F11-2 treated by BEN was measured with fluorescence probe 3,3′-dipropylthiadicarbocyanine iodide (DiSC_3_(5)) [46]. Bacteria grown to stationary-phase were inoculated into 5 mL LB broth at 37 °C with 200 rpm shaking for 8 h. Cultures were washed three times with PBS and then the DiSC_3_(5) was added at a final concentration of 2 μM. Bacterial suspension was incubated with 200 rpm at 37 °C for 30 min, 180 μL samples were transferred in a black-walled plate and fluorescence was measured immediately in an Infinite M200 Microplate reader (Tecan) with an excitation wavelength of 622 nm and emission wavelength of 670 nm. DiSC_3_(5) measurement was obtained at every 2 min for 32 min and the different concentration of BEN was added at the 8th minute.

### 4.7. Efflux Pump Assay

The fluorescence dye Rhodamine B [47] was used to evaluate the effect of BEN on the function of *tmexCD-toprJ*-mediated efflux pumps. Stationary-phase *K. pneumoniae* F105-1 and *P. mirabilis* F11-2 were washed three times with PBS and a final concentration of Rhodamine B (5 μM for *K. pneumoniae* F105-1 and 20 μM for *P. mirabilis* F11-2) was added. Next, cultures were placed in an incubator with 200 rpm at 37 °C for 30 min. After washing with PBS three times, the bacterial suspension was centrifuged (4000× *g*, 5 min) and resuspended in PBS containing 1% glucose. A different concentration of the BEN solution was then added and incubated at 37 °C for 30 min. Finally, the bacteria were centrifuged at 4000× *g* for 10 min and then 200 μL was taken to determine the fluorescence intensity using Infinite M200 Microplate reader (excitation at 622 nm, emission at 670 nm).

### 4.8. Tigecycline Accumulation Analysis

The accumulation of TIG in *K. pneumoniae* F105-1 and *P. mirabilis* F11-2 was determined by LC-MS/MS analysis according to a previous report [48]. Firstly, overnight cultures of bacteria were inoculated in fresh LB broth and grown at 37 °C under continuous shaking at 200 rpm for 8 h to an optical density (OD_600_) of 0.5, then cells were pelleted by centrifuging at 12,000× *g* for 10 min and diluted into 10^10^ CFUs per mL by PBS; the suspension was aliquoted into 1.5 mL tubes. Next, TIG at the MIC concentration together with varying concentrations of BEN were added and bacteria were cultured at 37 °C with shaking at 200 rpm.

After 15 min, cultures were centrifuged at 12,000× *g* for 3 min. To lyse the samples, 300 µL water was mixed with each pellet and then placed in liquid nitrogen followed by heating in a water bath at 55 °C for three freeze-thaw cycles. To collect the supernatants, the lysates were pelted at 12,000× *g* for 3 min. Next, in order to completely lyse the bacteria, the residual debris were re-suspended in 100 µL of acetonitrile and pelleted. A 0.22 µm filter membrane was used to filter all supernatants. Finally, all supernatants were detected by an Agilent 1260 Infinity HPLC system combined with AB SCIEX QTRAP 6500 mass spectrometer (ABSciex, Foster City, CA, USA). The mobile phase contained 0.2% formic acid in water (A) and acetonitrile (B). The separation was experimented on a C18 column and the flow rate was 0.4 mL/min. The linear gradient was as follows: 0.1–0.5 min, 80% A; 0.5–0.55 min, 80–10% A; 0.55–4.0 min, 10% A; 4.0–4.5 min, 10–80% A; 4.5–6.0 min, 80% A. The quantification detection of TIG accumulation was analyzed by multiple reaction monitoring (MRM) with positive electrospray ionization using the *m*/*z* 586.4→513.3 transition.

### 4.9. Galleria mellonella Infection Model

*Galleria mellonella* larvae (purchased from Huiyude Biotech Company, Tianjin, China) were divided into serial groups (*n* = 8 per group). All larvae were infected with 10^5^ CFUs *K. pneumoniae* F105-1 or 10^4^ CFUs *P. mirabilis* F11-2 suspension. At 1 h post-infection, infected larvae were treated with PBS, TIG (20 mg/kg), BEN (20 mg/kg), or the combination of TIG with BEN (20 + 20 mg/kg). Survival rates of *Galleria mellonella* larvae were recorded for 5 days.

## 5. Conclusions

In conclusion, we find that BEN greatly restores the TIG activity against TMexCD-TOprJ-positive pathogens both in vitro and in animal models of infection. To the best of our knowledge, this is the first report of a new TIG adjuvant in the fight against TMexCD-TOprJ-expressing microorganisms. Meanwhile, the addition of BEN also effectively thwarts the evolution of TIG resistance. These data suggest that BEN is a promising antibiotic adjuvant for combating novel RND efflux pump-conferred TIG resistance together with tigecycline. More preclinical studies are warranted to comprehensively explore the therapeutic potential of this drug combination.

## Figures and Tables

**Table 1:** Susceptibility profiles of Gram-negative bacteria harboring *tmexCD-toprJ* and potentiation of benzydamine to tigecycline.

Strains	MIC (μg/mL) ^a^									
				Tetracyclines						
	MEM	CIP	CL	MIN	OXY	DOX	TET	TIG	TIG ^b^	BEN
***K. pneumoniae***										
RGB 7-1	≤0.25	16	≤0.25	128	>256	64	>256	32	0.125 (256)	500
RGF 85-1	≤0.25	8	≤0.25	64	>128	64	>128	32	4 (8)	500
RGF 105-1	≤0.25	8	≤0.25	256	>256	64	>256	32	0.125 (256)	500
RGF 140-1	≤0.25	8	≤0.25	128	>128	128	>128	32	8 (4)	500
RGF 172-1	≤0.25	32	≤0.25	256	>256	256	>256	32	8 (4)	500
RGT 5-2	≤0.25	4	≤0.25	64	>128	64	>128	32	0.25 (128)	500
RGT 31-2	≤0.25	4	0.5	128	>256	128	>256	32	0.125 (256)	500
RGT 34-2	≤0.25	2	≤0.25	64	>256	256	>256	32	4 (8)	500
RGW 5-1	≤0.25	16	≤0.25	64	>256	128	>256	32	0.25 (256)	500
SZP 4-3-1	0.5	64	≤0.25	>256	>256	256	>256	32	16 (2)	500
TF 18-2	≤0.25	>128	≤0.25	>256	>128	128	>128	32	0.5 (64)	500
TF 44-1	≤0.25	≤0.25	≤0.25	>256	>128	64	>128	32	0.5 (64)	500
***P. mirabilis***										
RGF 11-2	≤0.25	64	>128	>256	>256	256	256	16	4 (4)	2000
RGF 134-1	≤0.25	64	>128	128	>256	128	128	32	4 (8)	2000

^a^ MEM, meropenem; CIP, Ciprofloxacin; CL, colistin; MIN, minocycline; OXY, oxytetracycline; DOX, doxycycline; TET, tetracycline; TIG, tigecycline; BEN, benzydamine. ^b^ MIC of tigecycline in the presence of 125 μg/mL (*K. pneumoniae*) or 500 μg/mL (*P. mirabilis*).

## Data Availability

Data is contained within the article.
